# High-resolution three-dimensional blood flow tomography in the subdiffuse regime using laser speckle contrast imaging

**DOI:** 10.1117/1.JBO.27.8.083011

**Published:** 2022-03-31

**Authors:** Chakameh Z. Jafari, Samuel A. Mihelic, Shaun Engelmann, Andrew K. Dunn

**Affiliations:** a The University of Texas at Austin, Department of Electrical and Computer Engineering, Austin, Texas, United States; b The University of Texas at Austin, Department of Biomedical Engineering, Austin, Texas, United States

**Keywords:** blood flow tomography, laser speckle contrast imaging, perturbation Monte Carlo, large-scale inverse problem, convex nonlinear optimization, stochastic gradient descent, advanced optimization

## Abstract

**Significance:** Visualizing high-resolution hemodynamics in cerebral tissue over a large field of view (FOV), provides important information in studying disease states affecting the brain. Current state-of-the-art optical blood flow imaging techniques either lack spatial resolution or are too slow to provide high temporal resolution reconstruction of flow map over a large FOV.

**Aim:** We present a high spatial resolution computational optical imaging technique based on principles of laser speckle contrast imaging (LSCI) for reconstructing the blood flow maps in complex tissue over a large FOV provided that the three-dimensional (3D) vascular structure is known or assumed.

**Approach:** Our proposed method uses a perturbation Monte Carlo simulation of the high-resolution 3D geometry for both accurately deriving the speckle contrast forward model and calculating the Jacobian matrix used in our reconstruction algorithm to achieve high resolution. Given the convex nature of our highly nonlinear problem, we implemented a mini-batch gradient descent with an adaptive learning rate optimization method to iteratively reconstruct the blood flow map. Specifically, we implemented advanced optimization techniques combined with efficient parallelization and vectorization of the forward and derivative calculations to make reconstruction of the blood flow map feasible with reconstruction times on the order of tens of minutes.

**Results:** We tested our reconstruction algorithm through simulation of both a flow phantom model as well as an anatomically correct murine cerebral tissue and vasculature captured via two-photon microscopy. Additionally, we performed a noise study, examining the robustness of our inverse model in presence of 0.1% and 1% additive noise. In all cases, the blood flow reconstruction error was <2% for most of the vasculature, except for the peripheral vasculature which suffered from insufficient photon sampling. Descending vasculature and deeper structures showed slightly higher sensitivity to noise compared with vasculature with a horizontal orientation at the more superficial layers. Our results show high-resolution reconstruction of the blood flow map in tissue down to 500  μm and beyond.

**Conclusions:** We have demonstrated a high-resolution computational imaging technique for visualizing blood flow map in complex tissue over a large FOV. Once a high-resolution structural image is captured, our reconstruction algorithm only requires a few LSCI images captured through a camera to reconstruct the blood flow map computationally at a high resolution. We note that the combination of high temporal and spatial resolution of our reconstruction algorithm makes the solution well-suited for applications involving fast monitoring of flow dynamics over a large FOV, such as in functional neural imaging.

## 1 Introduction

The ability to visualize longitudinal hemodynamics is essential in understanding the biological function and physiological progression in diseases affecting the brain such as stroke and Alzheimer’s and other neurodegenerative disorders.^1^ Optical imaging methods have had a significant impact in the field of neuroimaging and have been widely used to study the functional, cellular, and vascular physiology of the brain during disease states, particularly in animal models.^2^^,^^3^ These optical imaging modalities can be broadly generalized into two categories: macroscopic and microscopic.^4^

Optical imaging methods based on dynamic light scattering (DLS), such as laser speckle contrast imaging (LSCI),^5^ laser Doppler imaging,^6^ and diffuse correlation spectroscopy (DCS),^7^^,^^8^ provide blood flow maps over a large field of view (FOV) with resolution in the hundreds of microns to millimeters (macroscopic regime). LSCI’s advantage lies in its ability to rapidly image blood flow over a large FOV with the high spatiotemporal resolution, requiring relatively simple and cost-effective instrumentation. However, under traditional widefield illumination, its scope has been limited to providing volume integrated, two-dimensional maps of blood flow.

DCS, on the other hand, uses complex instrumentation of point source illumination and detection combined with photon diffusion models to sample deep in tissue. It uses a model-based computational tomography in the diffusion regime, assuming homogeneous structures with vascular volume fraction (VF), to derive a topographical blood flow index (BFI)^9^ with resolution limited to hundreds of microns. Additionally, DCS suffers from low signal-to-noise ratio (SNR) and low dynamic range since intensity autocorrelation $\left(g_{2}(t)\right)$ must be computed by measuring each speckle independently using a single or few-mode fiber.^10^ To increase the SNR, a large number of detectors (fibers) must be utilized at a given spot, making it either too complex or not feasible for practical DCS applications.

Most recently, LSCI has been extended to a model-based tomographic imaging paradigm using principles of speckle contrast imaging and photon diffusion.^10^^–^^12^ Similar to DCS, point-source illumination is used for model-based three-dimensional (3D) reconstruction. In speckle contrast optical tomography (SCOT) a high-density camera array replaces the complex detection instrumentation required in DCS, simplifying the instrumentation while improving the SNR. However, in both DCS and SCOT resolution is limited to hundreds of microns. Additionally, both these methods assume first-order approximations in the photon diffusion model as well as homogeneity assumptions in the tissue to solve the 3D reconstruction inverse problem analytically. In SCOT, additional assumptions must be made relating the observed speckle contrast to the underlying intensity fluctuations.^10^ We have shown previously that these assumptions lead to large errors in deriving decorrelations times used to estimate BFI in the subdiffusion regime, thus limiting the resolution and resulting in either under or overestimation of the vascular blood flow in a complex tissue.^13^

In the macroscopic regime, other noncoherent optical imaging modalities such as spatial frequency domain spectroscopy (SFDI)^14^^,^^15^ have recently been proposed, which have been shown to be capable of real-time imaging of optical properties of turbid media such as in the brain tissue. Zhao et al.^14^ presented a fast, real-time, noncontact, and label-free method for monitoring hemodynamics in a rat brain cortex over a large FOV based on principles of halftone SFDI. Although the latest advances in SFDI use simple instrumentation and achieve real-time imaging over a large FOV, their resolution is limited, and flow field maps lack 3D specificity.

On the other hand, in the microscopic domain, imaging modalities such as multiphoton microscopy (2/3PM)^16^ and to some extent optical coherence tomography (OCT)^17^ have enabled imaging down to a single neuron or capillary level with resolutions in a few microns. In the case of two-photon microscopy (2PM), high-resolution structural and functional images can be achieved with the ability to quantitatively measure blood flow down to a single capillary level. However, this imaging modality is often very slow, and its FOV is limited to only a few millimeters in each imaging session.

OCT enables imaging a larger FOV relative to 2PM, while keeping the resolution down in the few microns range.^17^^–^^19^ However, unlike 2PM, OCT does not provide the ability to quantitatively measure blood flow in capillary beds.^17^ Traditional Doppler OCT or OCT angiography methods have less sensitivity to vasculature in the transverse plane where capillary networks are mostly observed.^4^ Various techniques have been proposed to enable capillary velocimetry in OCT.^4^^,^^18^^,^^20^ However, these methods are limited in their dynamic range and do not allow for accurate concurrent measurement of blood flow in both capillary bed and arterial vasculature. Additionally, single-photon scattering is assumed in the derivation of the analytical models, which break down in the presence of multiple scattering photons in complex cerebral tissue.^20^

In this paper, we propose an innovative computational method for high-resolution tomographic reconstruction of blood flow maps in complex cerebral tissue down to capillary levels, using principles of speckle contrast imaging and perturbation Monte Carlo (PMC).^21^^–^^23^ It is important to note that our reconstruction algorithm requires the 3D geometry to be known or assumed to achieve the stated high-resolution reconstruction of blood flow. However, once the structure is imaged at a high resolution, the same geometry can be utilized to reconstruct blood flow maps over a large FOV rapidly while maintaining a high resolution down to the capillary level. This makes our proposed method particularly well suited for imaging blood flow over large fields of view.

Our proposed method utilizes simple instrumentation of point source illumination and camera array detection, similar to SCOT, to reconstruct 3D blood flow maps for a structural prior. However, unlike SCOT, in our reconstruction, we do not make any approximations or assumptions with respect to the particle flow dynamics or tissue homogeneity. Instead, we use perturbation of the simulated photon trajectories directly through the intact geometry to achieve high-resolution reconstruction of blood flow maps, noninvasively.

We previously showed the effect of vascular structure and homogeneity assumptions on blood flow estimates.^13^ Briefly, our results showed that randomizing vascular structure and defining a blood volume measure, rather than accounting for intact vascular structure, can lead up to 60% errors in electric field decorrelation times, even when considering measures relative to baseline. This leads to erroneous blood flow estimates, particularly in the subdiffuse regime, when comparing the blood flow in the vessel and the parenchyma regions.

Because Monte Carlo (MC) simulations solve the radiative transport equation (RTE) directly, in many studies, it is considered the gold standard for modeling photon migration in biological tissues.^24^^,^^25^ Nonetheless, MC was not deemed practical for providing the forward solutions due to its high computational overhead when simulating large volumes and limitations in modeling complex tissue structures. These limitations have been alleviated by implementation of high-resolution voxelized geometries^26^ and mesh-based MC methods,^27^^,^^28^ as well as efficient PMC methods to speed up the forward computation.^23^^,^^29^^,^^30^ Additionally, the wide availability of fast processors, such as graphical processing units (GPU) and super computers, has enabled the massive parallelization of the forward computation, thus reducing the processing time by several orders of magnitude. Recently, these innovations have led to an increased interest in using MC methods in a variety of fields for simulating the RTE forward model *in lieu* of the simplified analytical models.^21^^,^^30^^,^^31^

Our proposed 3D reconstruction framework is based on 3D DLS-MC,^32^ where the forward model and calculations of the Jacobian matrix are efficiently implemented in numerical MC perturbations. In DLS-MC, the aim is to efficiently evaluate the effect of a small change in the flow perturbation on the observed speckle contrast. This is achieved by reusing the trajectories of photons from an unperturbed simulation such that the trajectories do not need to be regenerated for each perturbation, resulting in reduced processing times.^23^^,^^32^^,^^33^

Given a structural prior, we present a novel, robust nonlinear optimization method for high-resolution reconstruction of blood flow map in complex cerebral tissue. We were able to overcome the low sensitivity of speckle contrast values to deeper vasculature by advanced optimization algorithms, enabling accurate reconstruction of the capillary flow down to 500-μm depth and beyond.

## 2 Theory and Methods

### 2.1 PMC-Based Forward Model

The underlying principle in LSCI is the relationship between the second moment of the electric field autocorrelation $\left(g_{1}(t)\right)$, when a coherent laser beam is an incident upon turbid tissue surface, and the observed speckle contrast (K) from the time-integrated back-scattered field, captured through a camera. Equation (1) formulates this relationship, where K is calculated experimentally as spatial variance $\sigma_{s}$ to the mean intensity ⟨I⟩ of a sliding 7×7 window swept across the image captured via a camera, for a given camera exposure time T. β is an instrumentation parameter and accounts for the mismatch between the detector and speckle spot size

$$K^{2}=\frac{\sigma_{s}^{2}}{{\left\langle I\right\rangle }^{2}}=\frac{1}{T}\int_{0}^{T}\beta {\left|g_{1}(t)\right|}^{2}(1-\frac{t}{T})dt.$$ (1)

We previously presented the details of our forward model platform, which is built on the theory of DLS-MC and aims to calculate the right-hand side of Eq. (1) accurately.^32^ In summary, when a plane wave electric field is incident on the surface of a medium, the resulting backscattered electric field at each detector has a phase shift that is the superposition of the momentum transfer contribution from each detected photon that underwent dynamic scattering due to interaction with moving red blood cells (RBCs) in vessels.^32^ The electric field autocorrelation function $\left(g_{1}(t)\right)$ can then be calculated according to Eq. (2) if the photon scattering position and vascular flow fields are known

$$g_{1}(t)=\langle E(0)E^{*}(t)\rangle =\int_{-\infty }^{\infty }P(Y)\exp(-2jk_{0}Yt)dY,$$ (2)

where t is the decorrelation lag time, P(Y) is the normalized length-dependent absorption weight for the detected photon, $k_{0}$ is the wavenumber, and Y is the dimensionless momentum transfer for each detected photon that underwent dynamic scattering (i.e., scattered inside a vessel at least once on its trajectory). The value of Y can be calculated according to the following equation:

Y=∑n=0N((k^f,n−k^i,n)·Vn), (3)

where ${\hat{k}}_{f,n}$ and ${\hat{k}}_{i,n}$ are the photon’s n’th scattering and incident unit vectors, respectively, and $V_{n}$ is the velocity vector of the corresponding scattering location inside the vessel.^13^ The sum is over all scattering locations for a single detected photon. The contribution of scattering in nonvascular regions to the momentum transfer Y is negligible, and thus $V_{n}$ is set to zero in the extravascular regions. We note that Eq. (3) captures only the ordered motion of RBCs in a vessel; however, it does not make any assumptions in terms of a number of scattering or degree of correlation.

In analytical solutions to Eq. (1), a form of $g_{1}(t)$ is assumed based on the dynamics of the particle flow by relating K to the electric field decorrelation times and inferring a blood flow measurement. Bandyopadhyay et al.^34^ formulated this relationship for different kinds of motion. In a complex tissue such as in the brain, where a single assumption in terms of particle dynamics is not valid, such simplifications can significantly limit the accuracy and resolution of the estimated blood flow measure. Our DLS-MC forward foregoes making any assumptions with respect to the tissue structure or the type of scattering and thus enables us to calculate the observed K values based on an accurate tissue model and flow structure.

### 2.2 Inverse Problem Formulation

For a set of illumination point sources ($N_{s}$) and detectors ($N_{d}$), the reconstruction algorithm can be formulated as a least-square minimization that seeks to reconstruct the blood flow map in tissue by minimizing the error between the observed speckle contrast through a camera and the estimated speckle contrast derived through our DLS-MC forward

V^=arg minvn∑Ns∑Nd‖Kmeasured−1T∫0Tβ|g1(t)|2(1−tT)dt‖2+γR(V^). (4)

In this formulation $N_{s}$ is the number of geometry-dependent point source illuminations spots scanned across the geometry surface, and $N_{d}$ is the number of detectors resembling a camera detector array. V^=[v^1,v^2,v^3,…,v^N]T, where $v_{i}$ is the estimated blood flow in vessel strand i. We note that a strand object is any continuous vessel segment between two bifurcation points derived through vectorization of the geometry as further discussed in Sec. 2.5. K is the measured or simulated speckle contrast values based on ground truth vascular flows. In calculating the momentum transfer in Eq. (3), all unit vector directions of photon scattering, and vascular centerlines are known, and the inversion reconstructs the scalar component of ${\overset{\to }{V}}_{n}$, namely the blood flow in the corresponding strand.

In Eq. (4), the data fidelity term ${\left\|.\right\|}^{2}$ represents an $\ell_{2}$ norm. The regularization term, $\left(R(\hat{V})\right)$ imposes a prior on the reconstructed vascular flow with tunable regularization parameter γ to avoid overfitting due to the high degree of nonlinearity of our model and to reduce noise artifact subject to positivity constraint. As further discussed in Sec. 2.3, a one-dimensional total variation (TV1D) regularization R(V^)=‖V^‖TV1D was implemented, as it provides sufficient regularization while balancing speed and memory overhead.

### 2.3 Derivation of the Jacobian Matrix

The first step in solving the minimization problem presented above is to compute derivatives with respect to the vascular flow profiles. While the data misfit term in Eq. (4) $\left({\left\|.\right\|}^{2}\right)$ is differentiable, the regularization term may be smooth but not differentiable depending on the type of prior. In such cases, iterative proximal stochastic gradient methods present lower complexity in terms of memory requirement and computational overhead when compared with alternating direction method of multipliers (ADMM) or second-order Newton’s method^35^ while achieving a fast convergence rate. Herein, we present the analytical expression for the PMC-based derivative of the data misfit term and discuss the choice of regularization function and the proximal operator for computing the Jacobian matrix.

Starting from the complex-valued expression of $g_{1}(t)$ from Eq. (2), the absolute value of the second moment ${\left|g_{1}(t)\right|}^{2}$ can be rewritten as

$${\left|g_{1}(t)\right|}^{2}={\left|\overset{N-1}{\underset{n=0}{\sum }}P_{n}e^{-j2k_{0}Y_{n}t}\right|}^{2}=\overset{N-1}{\underset{n=0}{\sum }}P_{n}^{2}+\overset{N-2}{\underset{n=0}{\sum }}\overset{N-1}{\underset{m=n+1}{\sum }}2P_{n}P_{m}\;\cos(2k_{0}(Y_{n}-Y_{m})t).$$ (5)

In this expression, the sum is over all the photons detected by a single camera pixel (detector), $P_{n}$ and $P_{m}$ are the normalized length dependent photon weight for photons n and m. $Y_{n}$ and $Y_{m}$ are the momentum transfers calculated according to Eq. (3) at a given point in search space for photons n and m. Using Eq. (5), the derivative of the data misfit with respect to individual strand vascular flow can then be calculated as follows:

∂‖K−K^‖l22∂vi=(KK^−1)∑n=0N−2∑m=n+1N−1∂K^nm∂vi, (6)

where

∂K^nm∂vi=2(qn−qm) sin(k0TA)((k0T)2A3)(k0TA cos(k0TA)−sin(k0TA)). (7)

In Eq. 7 ${\hat{K}}_{nm}$ is the speckle contrast estimate accounting for only photons n and m detected by a given detector, and A is their net momentum transfer contribution $Y_{n}-Y_{m}$ derived from Eq. (3). $q_{n}$ and $q_{m}$ are the unit dot products of photon scattering and vessel centerline direction at location r, if r is in the subspace of strand object i. If photons n or m have not been dynamically scattered in vessel i, then their corresponding q value will be set to zero.

A proximal operator can be used in computing the derivative for Eq. (4) when the regularization function (R) is not differentiable. In our tissue vectorization step, all strand objects are sorted according to their radii size. We note that $V\in R^{3}\to R$ is the projection of strand objects in 3D space onto neighboring strands of similar radii and flow in vector form. In this paper, we have chosen TV1D as the penalty function since it has an efficient proximal operator and provides reasonable regularization.^36^^,^^37^ The following proximal operator is applied after the gradient step to address the illconditioning of our highly nonlinear problem

proxγR(V^)=arg minx(12‖x−V^‖ℓ22+γ‖x‖TV1D). (8)

Equation (8) is solved iteratively, applying regularization to x, while keeping the solution close to the updated $\hat{V}$ derived in the gradient step. Care must be taken in tunning the regularization parameter (γ) so that large variation in neighboring strand objects is not blurred out while sufficient regularization is applied to noisy reconstructed data.

### 2.4 Reconstruction Framework

Algorithm 1 illustrates our mini-batch gradient descent optimization method implemented for PMC-based 3D reconstruction of vascular flow in a complex tissue. One of the challenges in reconstructing blood flow in deeper vascular regions is that the observed speckle contrast images have lower sensitivity to variations of blood flow deep in tissue. Photon sampling is highly concentrated toward the superficial vascular region.^38^ This compounds the choice of the learning rate for optimal convergence and accuracy in the gradient descent step.

**Algorithm 1:** 3D PMC-based blood flow tomography.

***Input:*** Measured or simulated speckle contrast images based on ground truth vascular flow (K), number of illumination points $N_{s}$ and total number of camera array pixels/detectors $N_{\text{dtotal}}$, Y, and P(Y) values calculated from postprocessing of photon trajectories, separated by scattering locations and binned by detector, maximum number of iteration $N_{\text{iter}}$, regularization parameter γ, adaptive moment estimation parameters $\beta_{1},\beta_{2},\eta ,\varepsilon$, and error tolerance etol
***Initialization:*** Vascular flow for the N strand objects initialized to 1 and the corresponding proximal values and gradients set to 0.
{V^1}i=1N=1,{V^prox,0}i=1N=0,{V^grad}i=1N=0,t0←1,m^,v^,c←0
1: **for** $k\leftarrow 1\text{ to }N_{\mathrm{iter}}$ **do**
2: **for** $j\leftarrow 1\text{ to }N_{s}$ **do**
3: $N_{\text{dbatch}}\leftarrow \text{random}.\text{sample}(N_{\text{dtotal}},\text{BatchSize})$ ▹ Distribute through MPI
4: **for** $m\leftarrow 1\text{ to }N_{\text{dbatch}}$ **do**
5: ${\mathrm{res}}_{jm}\leftarrow |K_{{\text{measured}}_{jm}}-\sqrt{\frac{1}{T}}\int_{0}^{T}\beta {\left|g_{1_{jm}}(t)\right|}^{2}(1-\frac{t}{T})|dt$
6: $\{{\hat{V}}_{{\mathrm{grad}}_{k}}{\}}_{i=1}^{N}\leftarrow \{{\hat{V}}_{{\mathrm{grad}}_{k}}{\}}_{i=1}^{N}+\frac{\partial {\mathrm{res}}_{jm}^{2}}{\partial v_{i}}$ ▹ PMC-Based [Eq. (6) or FD]
7: **end for**
8: **end for**
9: $c_{k}\leftarrow c_{k-1}+\underset{N_{\text{dbatch}}}{\sum }{\left|\mathrm{res}\right|}^{2}$
10: update first and second gradient moment $\left({\hat{m}}_{k},{\hat{v}}_{k}\right)$ ▹ Ref. 39
11. {V^k}i=1N←{V^k}i=1N−ηv^k+ε ▹ Adam update
12. {V^prox,k}i=1N=proxγR({V^k}i=1N) ▹ Regularization
13. $t_{k}\leftarrow \frac{1}{2}(1+\sqrt{1+4t_{k-1}^{2}})$
14. $\{{\hat{V}}_{k+1}{\}}_{i=1}^{N}\leftarrow \{{\hat{V}}_{\mathrm{prox},k}{\}}_{i=1}^{N}+\frac{t_{k-1}-1}{t_{k}}(\{{\hat{V}}_{\mathrm{prox},k}{\}}_{i=1}^{N})-\{{\hat{V}}_{\mathrm{prox},k-1}{\}}_{i=1}^{N}$ ▹ Acceleration [Nesterov]
15. **if** $c_{k}-c_{k-1}<\mathrm{etol}$ **=> exit**
16. **end for**
***Return:*** The reconstructed vascular flow in each strand {V^k+1}i=1N

In this paper, we have used gradient descent optimization algorithms based on adaptive learning rate estimates to alleviate the discrepancy in lower sampling frequency of deeper vascular regions. Adaptive moment estimate (Adam)^39^ adaptively adjusts the learning rates based on decaying averages of the first and second moments of gradients, $m_{t}$ and $v_{t}$.

In addition to the adaptive learning rate optimization algorithm, we have also utilized Nesterov’s momentum acceleration scheme^40^ which significantly improves our convergence rate. Finally, we implemented fast direct methods for calculation of the proximal operator for TV1D.^41^ Collectively, these schemes resulted in $O(1/\sqrt{\varepsilon })$ convergence rate, where ε is the desired error tolerance.

### 2.5 Geometry Vectorization and Simulation Platform

We previously reported on the details of our DLS-MC-based simulation platform used as the forward model in generating speckle contrast images.^13^ The same PMC-based model was utilized in calculating the forward model in each iteration of the gradient descent step. The structural prior, utilized in our reconstruction algorithm was obtained via 2PM imaging^42^ and was vectorized using the segmentation-less, automated, vascular vectorization method.^26^

Briefly, vascular objects were vectorized using 3D, multiscale, linear filtering of unprocessed image volumes. Vectorized vessel objects contain the volumetric centerline flow field and vessel radii information at each voxel. This rapid vectorization algorithm allows for the extraction of strand objects, providing a volumetric vascular connectivity map in addition to statistical information, such as VF and vascular morphology. The complete vectorized vascular network is partitioned into strand objects, which are defined as the 1D vessel traces between the bifurcation points and endpoints of the network. However, the large superficial or descending vessels often have densely spaced bifurcations where smaller branches connect, resulting in many small strand objects along these larger vessels. Accordingly, we implemented additional smoothing to avoid very short strand objects in these larger vessels by connecting the largest two strands at each bifurcation, yielding longer, more continuous “superstrand” objects that, like the strand objects, also partition the network into 1D traces between bifurcations and endpoints.

**Fig. 1:**
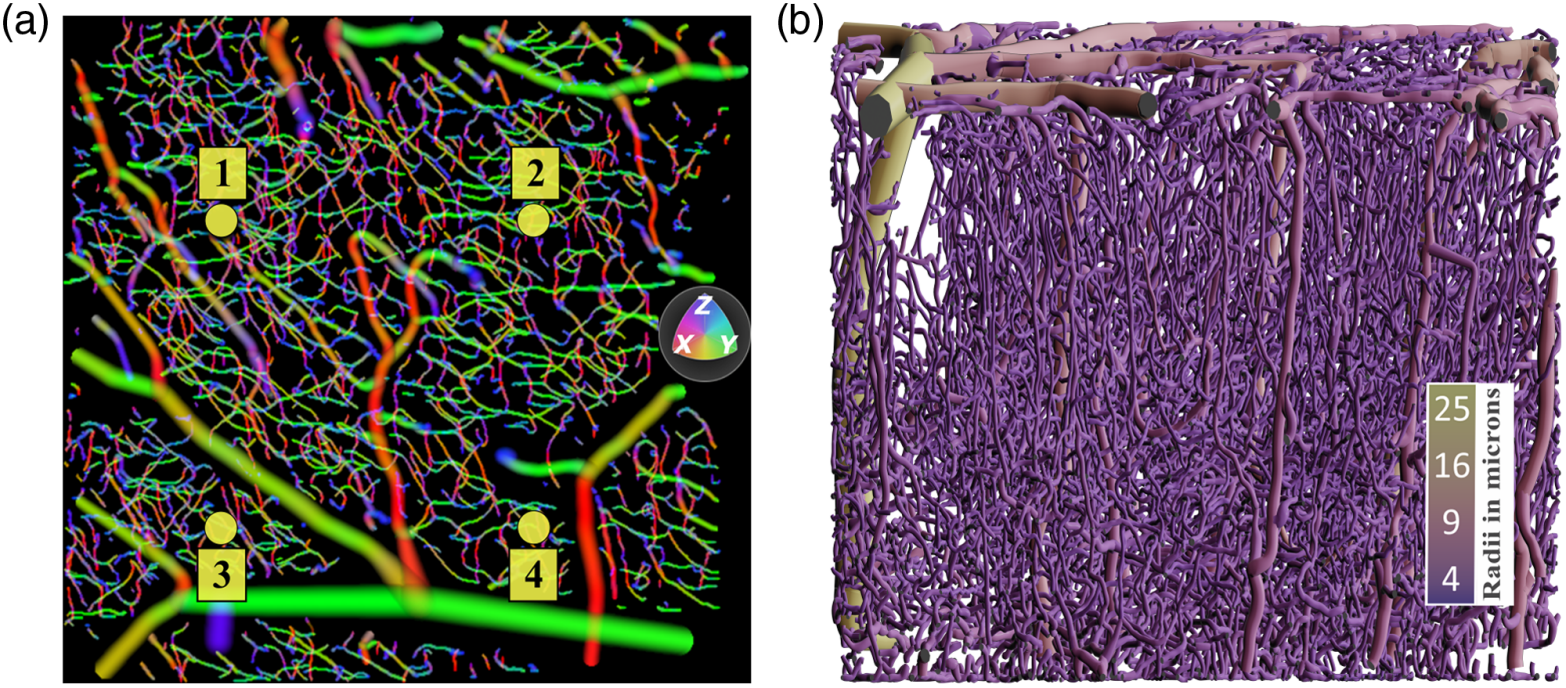
Sample geometry and illumination scheme used in our reconstruction algorithm. (a) X–Y projected vascular flow fields of murine cortex vasculature acquired via 2PM microscopy and vectorized through our vectorization platform. The vascular centerline directions are color-coded [(x,y,z) in (red, green, blue)] and laminar flow profiles evident in larger vessels. (b) Axial profile of the same vectorized geometry rendered in Blender^43^^,^^44^ color-coded based on vessel radii with larger surface vasculature in a green and capillary network in dark purple.

Figure 1 illustrates a sample vectorized geometry used in our reconstruction algorithm with dimensions 1.134×1.064×0.726  mm in the x, y, z directions, respectively. Figure 1(a) shows the transverse projected direction of the vasculature centerline flow fields with x, y, and z directions color-coded in red, green, and blue, respectively. Figure 1(b) shows the axial projected vascular structure of the same geometry color-coded based on vessel radii with larger surface vessels in light green and capillary network in dark purple.

Parallelized DLS-MC simulations were launched on the Stampede2 Skylake compute nodes on Texas Advanced Computing Center (TACC) using the message passing interface (MPI) protocol. Photon trajectories and absorption weights were simulated for the four different illumination points shown in Fig. 1(a). In each simulation, a collimated beam with a diameter of 40  μm was set to illuminate the geometry at the entrance position shown (yellow circle). We launched a minimum of $2\times 10^{9}$ photons in each simulation and for photons reflected through the top surface of the geometry we recorded all entry and exit locations as well as the photon trajectories through the volume and photon weights.^32^ Generation of $g_{1}(t)$ and speckle contrast values and normalized unit Y vectors from Eq. (3) (a scalar component of vascular velocity set to 1) for all the detected photons were implemented in Python and parallelized through MPI in a secondary postprocessing step.^13^ These simulations took approximately between 30 and 155 s on 200 cores of Stampede2 Skylake compute nodes on TACC for the geometries presented in this paper.

Our novel reconstruction algorithm which we have also implemented in Python utilizes the binned normalized Y and P(Y) values from the postprocessing step to reconstruct the blood flow maps in tissue iteratively according to Algorithm 1. The iterative algorithm was optimized to enable processing on both GPU and multicore processing through MPI for performance comparison. The calculation of Jacobian was heavily vectorized for GPU processing through efficient sparse matrix operations.

## 3 Simulation Results

We evaluated the performance of our reconstruction algorithm numerically through simulation of reconstructed flow maps for vascular phantom model and actual murine cortex vascular network captured through 2PM. The ground truth blood flow values were assigned based on radii size thresholds according to values reported in the literature^45^ and further discussed in Secs. 3.1 and 3.2. For each geometry, we ran two different sets of DLS-MC simulations to ensure the uniqueness of photon trajectories used in generating the ground truth images versus the reconstruction algorithm. The following sections discuss the geometry and demonstrate the robustness and accuracy of our reconstruction algorithm in the presence of noise even for deeper vasculature structures.

### 3.1 Reconstructed Phantom Flow Map

**Fig. 2:**
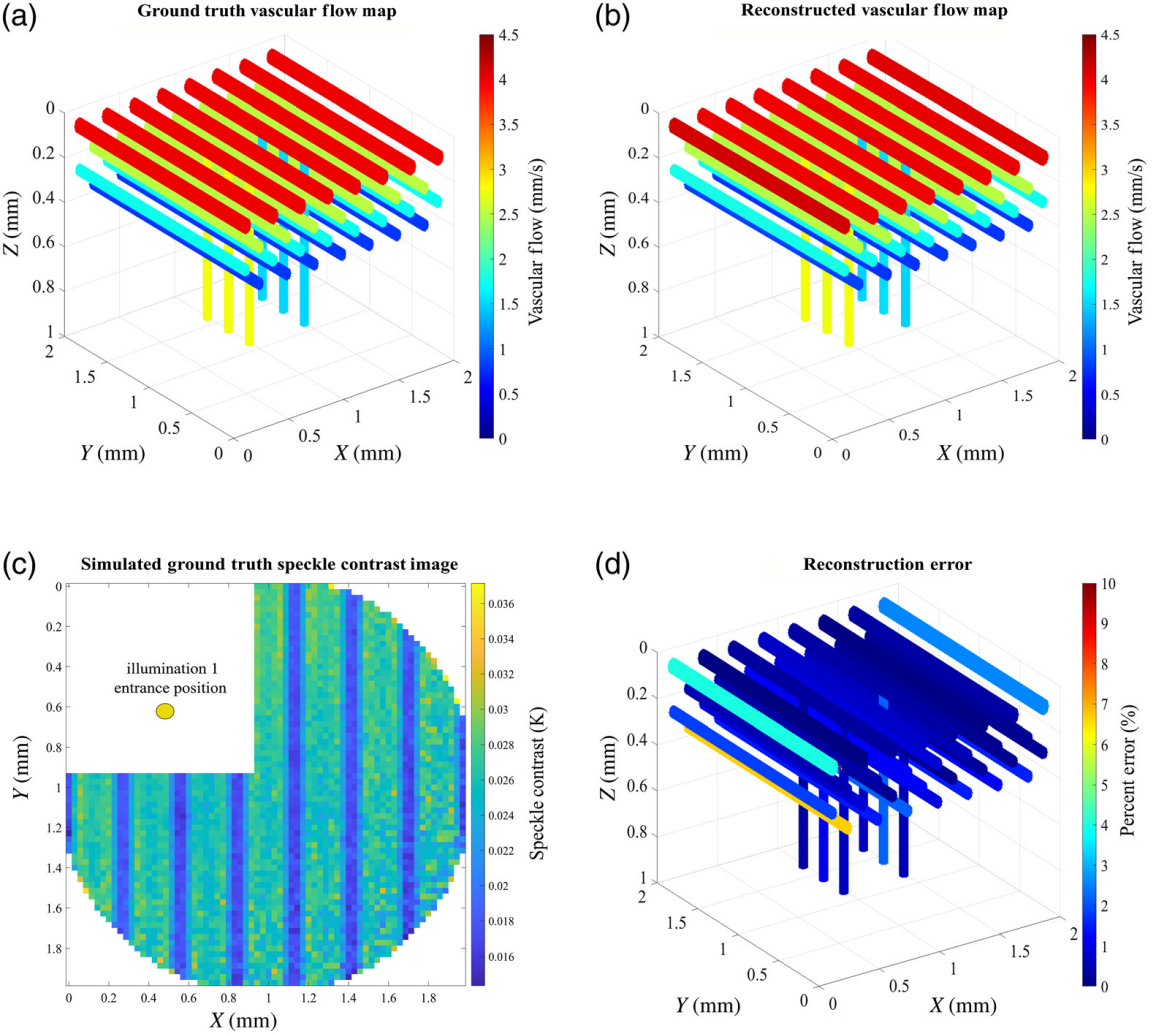
Illustration of the 3D blood flow map reconstruction accuracy through numerical simulation of vascular phantom. (a) Ground truth vascular flow map. (b) Illustration of the reconstructed vascular flow map after 160 iterations of the reconstruction algorithm. (c) Ground truth speckle contrast image for point source illumination 1. Speckle contrast images were generated for each of the illumination points shown in Fig. 1, resulting in four different ground truth simulated speckle images. Pixels within 200  μm of the source were excluded to prevent over saturation. Missing quadrant in the speckle contrast image illustrates the detectors that were excluded in the reconstruction algorithm under illumination 1 due to saturation. (d) Reconstruction accuracy (% error) after 160 iterations of the reconstruction algorithm, with mean error bound <2% and the highest error observed on the peripheral vasculature due to a low number of reflected photons in these regions.

Our vascular network phantom model is depicted in Fig. 2(a). The phantom includes a set of horizontal and descending vasculature with an overall size of 2×2×1  mm in the x, y, and z directions, respectively and 5-μm cubic voxels. The horizontal vasculature was interleaved within the axial layers and extended down to a depth of 500  μm. The vertical descending vasculature was placed normal to the surface of the geometry. They were defined as bifurcations immediately below the first layer vasculature and were separated by 500 and 200  μm in the x and y directions, respectively. The ground truth blood flow values were assigned randomly ranging from 0.3 to 5  mm/s.^4^ The optical properties for vascular and extravascular regions were set based on values we reported previously^32^ and specified here in Table 1 for completeness. A 100×100 array grid with detector size of 20  μm×20  μm was defined as the detection geometry. Once photon trajectories and absorption weights were simulated in the MC step, binning of the reflected photons for the large detector grid and computation of $g_{1}(t)$ and speckle contrast image in the post-processing step took ∼25  s on a total of 200 cores. The camera exposure time was set to 3 ms in calculating the speckle contrast images.

**Table 1:** Optical properties of vasculature geometry.

	$\mu_{a}$ (${\mathrm{mm}}^{-1}$)	$\mu_{s}$ (${\mathrm{mm}}^{-1}$)	g
Capillaries	0.2	65	0.98
Noncapillaries	0.2	90	0.98
Extravascular	0.02	10	0.9

Figure 2(c) shows a sample speckle contrast image for point source illumination 1. A ground truth speckle contrast image was generated for each of the illumination spots similar to the source positions depicted in Fig. 1(a), resulting in four different simulated images. The point sources were separated by 500  μm, covering the four quadrants of the geometry. Additionally, detectors within 200-μm radius of the point source were excluded in each case to avoid over saturation of the pixels. Figure 2(c) depicts the detectors that were excluded (missing quadrant) in the reconstruction algorithm due to saturation, under source illumination 1. The normalized Y and P(Y) values were stored in a binary file format for each of the $N_{\text{dtotal}}$ and $N_{s}$ detector and source pairs resulting in 33,600 total files available.

In the reconstruction algorithm, all strand object vascular flow values were initialized according to Algorithm 1. The mini-batch size was set to randomly sample 1/8 of the total detectors (with replacement) in each iteration of the gradient descent. Values of $\beta_{1}$, $\beta_{2}$, and η in Algorithm 1 were heuristically set to 0.8, 0.999, and 0.1, respectively. These values provided the best combination of convergence speed and accuracy across all tested geometries. The processing of detectors and calculation of the Jacobian matrix was distributed to 200 cores of Stampede2 Skylake compute nodes on TACC through MPI protocol. The phantom geometry included a total of 54 strand objects. Each iteration of the reconstruction algorithm took 4.5 s, with an aggregate reconstruction time of 12 min for all 160 iterations.

**Fig. 3:**
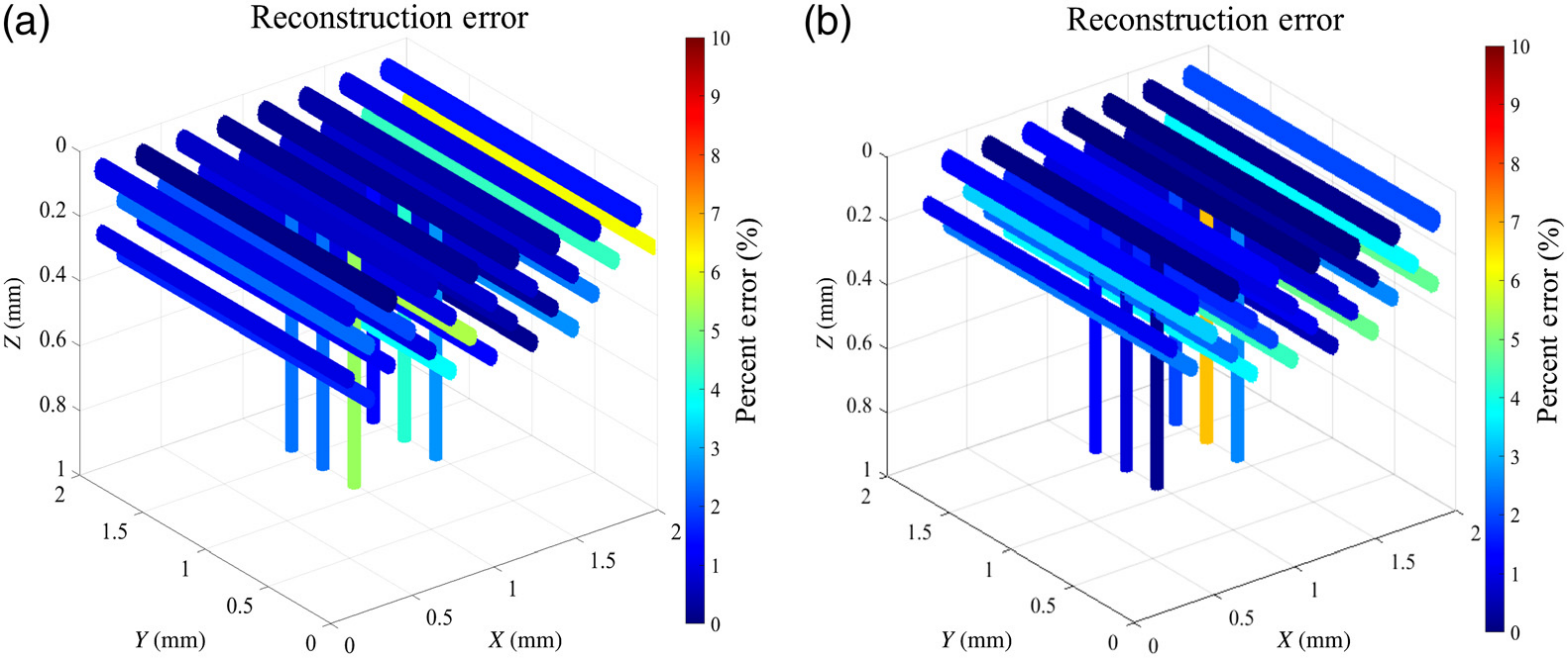
Analysis of reconstruction accuracy in presence of noise. (a) Reconstruction error subject to 0.1% additive noise. (b) Reconstruction error subject to 1% additive noise.

To evaluate the performance of our regularization scheme and to assess the robustness of our reconstruction algorithm in presence of noise, we performed a noise study analysis by perturbing the ground truth simulated speckle contrast images by 0.1% and 1% additive noise N(0,N), where N is the noise level. The additive noise was distributed across the 100×100 detector grid to generate noisy speckle contrast images to be used in the reconstruction algorithm. Figure 3 illustrates the reconstruction error in presence of noise for each of the noise levels.

### 3.2 Reconstructed Murine Cerebral Flow Map

**Fig. 4:**
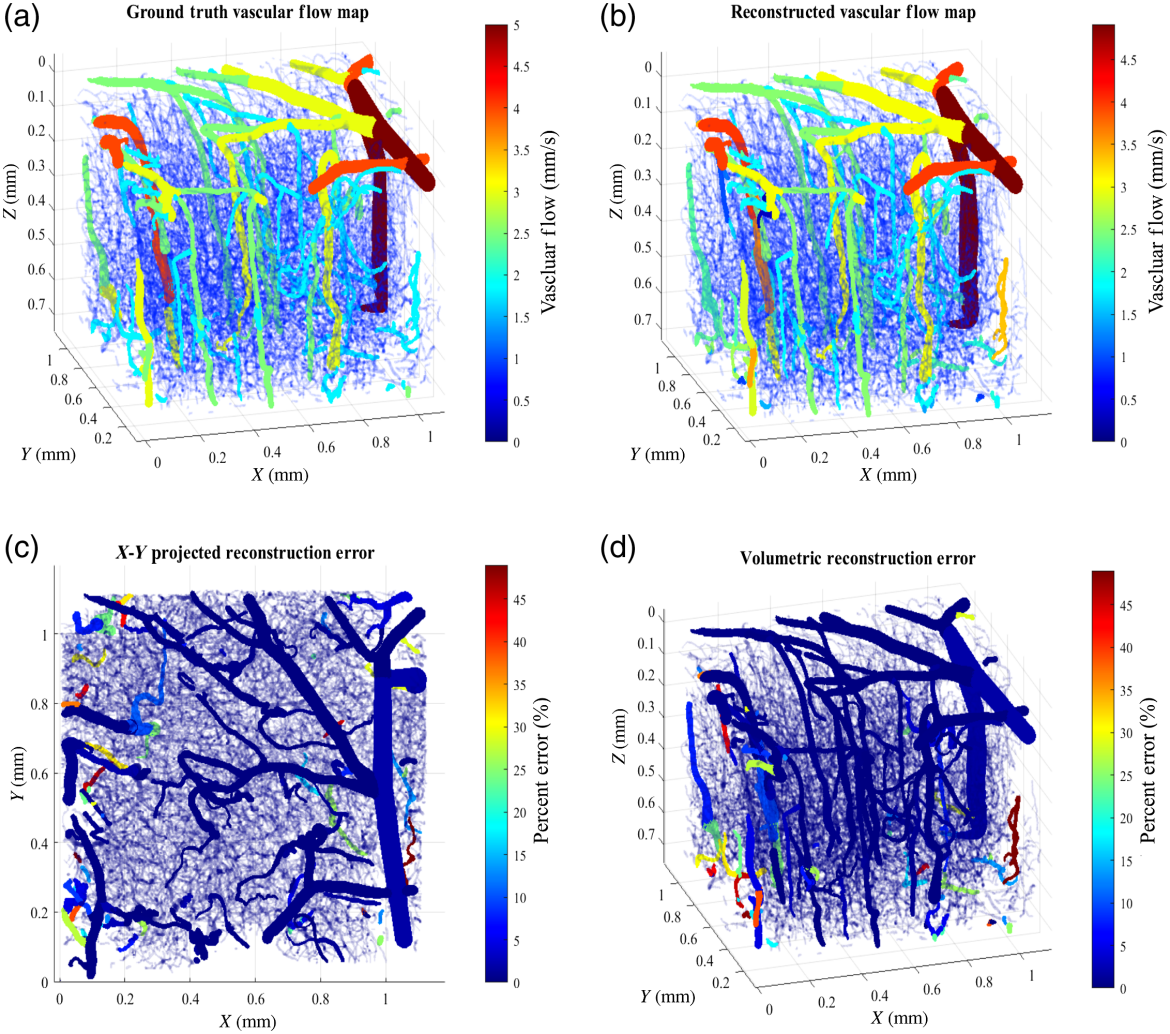
Illustration of the 3D blood flow map reconstruction accuracy through numerical simulation of murine cerebral tissue, captured via 2PM. (a) Volumetric illustration of the vascular flow values, assigned in simulating the ground truth speckle contrast images. (b) Reconstructed vascular flow map on the 200th iteration of the reconstruction algorithm. (c) Reconstruction error [percent error between (a) and (b)] projected on X–Y plane. (d) Volumetric demonstration of the reconstruction accuracy (error %) for the same iteration. The results show high fidelity reconstruction of flow in large and small vasculature of different orientations in an actual complex network. While reconstruction error in the majority of vasculature is limited to below 3%, deeper vasculature in the periphery shows higher error bounds due to the circular nature of detector geometry and a small number of reflected photons in these regions.

Figure 4 depicts the robustness and accuracy of our high-resolution 3D reconstruction algorithm on physiological complex cerebral tissue. A high-resolution vascular network of the murine cortex was obtained through 2PM imaging^42^ and vectorized as discussed earlier in Sec. 2.5. The geometry includes vasculature with radii ranging from 4  μm in the capillary network to 25  μm in the larger superficial and descending vasculature. All vessels with radii <5.5  μm were specified as a capillary network. In the DLS-MC simulation, the optical properties were assigned based on values we reported previously^32^ and specified here in Table 1. The ground truth centerline blood flow values in each strand object were set according to arterial, capillary, and venous radius-based velocities presented in the literature.^45^ These values ranged from 0.3  mm/s in the capillary network to 6  mm/s in larger superficial vasculature for the vascular structures present in this geometry.

Boas et al.^46^ presented a closed vascular anatomical network with a pressure-flow circuit model to derive accurate flow distribution in individual strands. They illustrated that the vascular flow distributions derived through the circuit model, follow that of the radii-based velocities validated experimentally.^45^ Since our vectorized vasculature sets are not closed networks, they do not allow for tracing and assigning direction in each strand and thus limit our ability to use the pressure-flow circuit model. As such, we used a radii-based lookup table to set the centerline velocities according to the values reported in Ref. 45. Additionally, flow directions were randomly assigned in the vectorization step. Given the nature of speckle contrast calculations, this assumption has minimal impact on the simulated forward model and thus will not affect the accuracy of our reconstructed results (please refer to the Supplemental Materials for further illustration).

We note that in our reconstruction algorithm, capillary networks within a $250\times 250\times 250\;{\mu m}^{3}$ cubic region were assumed to have the same underlying flow. This assumption, while valid given the density of arterial and venules in the geometry, significantly speeds up the convergence of our reconstruction algorithm by reducing the number of strand objects in the inverse problem.

All initialization and optimization parameters were set according to Algorithm 1 and the values reported in the phantom reconstruction section. The regularization parameter γ was set to $10^{-3}$, which was found to provide the best combination of regularization without blurring the adjacent vascular flows. An 80×80 array grid was defined as the camera detector geometry with a pixel size of $13\times 13\;{\mu m}^{2}$. Four illumination point sources were simulated in generating the ground speckle contrast images as described earlier in Sec. 2.5. Detectors within 200  μm of the source position were excluded in each simulation to avoid over saturating the pixels. The normalized Y and P(Y) valued for each of the $N_{\text{dtotal}}$ and $N_{s}$ detector and source pairs resulted in 19,824 total files available. The batch size was set to randomly sample 1/16 of the total detectors (with replacement) in each iteration of the gradient descent and the processing of the Jacobians was distributed through MPI protocol as reported previously. The shown geometry included a total of 1674 individual strand objects resulting in a 1674×1240 Jacobian matrix calculation in each mini-batch step. Each iteration of the reconstruction algorithm took 9.6 s, with an aggregate reconstruction time of 32 mins for all 200 iterations.

Figure 4 illustrates the reconstruction error (%) on the 200th iteration of the algorithm. As shown, our proposed algorithm reconstructs the vascular flow in most of the vasculature with an error bound <2%. This is especially true for the larger superficial and descending vasculature where the error is below 0.1%. We observed larger errors in smaller vascular regions on the periphery where the error could reach as high as 40%. This can be explained given the circular nature of our detector geometry in the DLS-MC step resulting in very few photons reflected in this region.

## 4 Discussion

### 4.1 Analysis of the Reconstruction Accuracy for the Simulated Geometries

Our simulation results demonstrate the high-fidelity reconstruction of blood flow map in complex tissue with resolution down to individual vessel and capillary strand objects, beyond 500-μm depth. As previously discussed, we observed an error bound below 2% on the reconstructed flow estimates for both the vascular phantom model and the physiological murine cortex tissue. In particular, this value was lower for larger superficial vasculature, where the error was within 0.1%.

In both cases, the reconstruction error in the peripheral vascular strands was significantly higher than the rest of the geometry. We believe there are two explanations for this. First, we note that the detection geometry in our DLS-MC step is defined as a circular region, mimicking the circular aperture of a camera. Given that our geometry is cubic, this limits the number of detected photons that would have otherwise sampled the vasculature in the periphery and reflected through the corners. Second, the vascular objects in the periphery reside at the tissue boundary, where the probability of photons exiting in the plane normal to the boundary is higher, resulting in fewer reflected photons through the surface in these regions. The combination of these two phenomena results in a much lower sampling of the peripheral vasculature, which in turn leads to very small gradients in each iteration of the reconstruction algorithm. While the error in the peripheral vasculature is expected to decrease with a large number of iterations, we note that the peripheral vascular regions should perhaps be excluded after reconstruction to maintain reasonable reconstruction times.

### 4.2 Noise Analysis

Figure 3 shows the performance of our reconstruction algorithm in the presence of noise. As demonstrated, the reconstruction error remains low (<6%) across the geometry, as noise levels in the simulated speckle contrast images are raised. This value is lower (<2%) for superficial horizontal vasculature with higher noise sensitivity observed only in deeper vasculature beyond 300  μm. The descending vasculature shows slightly higher fluctuations in presence of noise when compared with horizontal vessels. This can be explained in part through our choice of geometry and detector grid. The discrepancy in the number of detectors sampling the horizontal vessels, as opposed to the vertical vasculature for this phantom leads to less averaging of noise for detectors sampling vertical vessels thus resulting in higher susceptibility to noise. Our noise analysis illustrates the robustness of our regularization scheme in preventing over-fitting of the data in presence of noise, despite the illposed nature of our simplified phantom. As a next step, we will be examining a regularization scheme based on the log-likelihood of the vascular covariance matrix, imposing both vessel size and connectivity as a prior to better represent the physiological structure and to further improve accuracy.

### 4.3 GPU Processing Optimization

We optimized our calculations to enable efficient GPU processing and compared the reconstruction performance against our results obtained through distributed multicore processing. To gain significant speed up on GPU the forward model and calculation of the Jacobian matrix had to be heavily vectorized.

We note that the calculation of $g_{1}(t)$ can be cast as sparse matrix multiplication of the form $\exp(-2jk_{0}V^{T}Q)P$. In this formulation, $V^{T}$ is the vector of blood flow maps of the size 1×N, where N is total number of strand objects in the geometry. $Q_{NXM}$ is a sparse matrix where each entry is the normalized Y values from Eq. (3) for photon m and strand object n. Given that each photon only samples a few strand objects on its trajectory, each column of the Q matrix will only include a few nonzero entries. P is a vector of absorption weights and has a size M×1. We followed the same procedures in converting all derivative calculations to sparse matrix operations to enable efficient vectorization on GPU. This formulation, in conjunction with efficient sparse matrix operations in python, allowed for significant speedup of reconstruction when using GPU for processing. Our results indicate that simulation time for each iteration of the mini-batch gradient step took ∼3× longer to run on our Nvidia GTX processor when compared with 200 cores of Skylake compute nodes on TACC. However, simulation times could be significantly larger on GPU when matrix sizes become too large and GPU memory capacity issues are encountered. As such, multicore processing is preferred when possible since it is proven to be a fast and robust method even when considering a large number of strands.

### 4.4 Analytical versus Finite Difference Calculation of the Jacobian Matrix

In Sec. 2.3, we derived the analytical expression for calculating the derivative of the speckle contrast at a given detector with respect to blood flow in an individual strand object. The finite difference (FD) calculation of the derivative with respect to flow in each strand object can be formulated as $\frac{\partial K_{m}}{\partial v_{i}}=\frac{K_{{\mathrm{res}}_{m}}(K_{m}(\hat{V},{\hat{v}}_{i}+\varepsilon )-K_{m}(\hat{V},{\hat{v}}_{i})}{\varepsilon }$, where $K_{m}$ is the forward model according to Eq. (1) for a given detector m, at a point in search space $\hat{V}$, with an asymptotically small perturbation ε. $K_{{\mathrm{res}}_{m}}$ is calculated according to line 5 in Algorithm 1. We note that in most inverse problems, the calculation of derivatives through FD is cost-prohibitive as it requires two forward calculations for each parameter. We showed in Sec. 4.3 that our PMC-based forward calculation of $g_{1}(t)$ can be cast as a sparse matrix multiplication resulting in a fast calculation of the forward model. We can further vectorize the derivative calculations with respect to all flow parameters by implementing a sparse matrix multiplication of the following form:

∂Km∂V^=Kresm(exp⁡(−2jk0(V^+Iε)TQ)−exp⁡(−j2k0V^Q))Pε. (9)

In this formulation, I is the identity matrix, (V^+Iε)T is an N×N matrix, where N is number of strand objects. Q and P are as described in Sec. 4.3.

According to Eq. (6), the analytical derivative calculation requires an M×(M−1) matrix operation, where M is the number of detected photons at a given detector and ranges between 1000 and 4000 depending on the location of the detector. Our results show that the FD method provides a faster calculation when inverting for a smaller number of parameters, similar to our simplified phantom model. Additionally, the vectorized formulation of Eq. (9) is better suited for GPU processing; however, care must be taken not to exceed GPU memory capacity when the number of strand objects becomes large. Calculation of the derivative with respect to all parameters at a single detector point took 0.65 ms using the FD method as compared with 0.72 ms through the analytical expression for our murine geometry described in Sec. 3.2. However, as the number of strand objects in the geometry becomes large, the analytical expression of the derivative calculation becomes more feasible.

### 4.5 Limitations and Future Work

Although our proposed method has shown to be effective in reconstructing blood flow maps in tissue at a high resolution, there are some limitations that require further exploration. One of the main limitations of our proposed method is that it requires a correct 3D geometry model for high-resolution reconstruction of blood flow map in a complex tissue. However, the 3D structure needs to be captured only once and can be reused subsequently in the reconstruction algorithm for longitudinal studies. As described in Sec. 2, once the 3D structure is imaged at a high resolution, the experimental setup for capturing the speckle images used in the reconstruction algorithm, involves scanning the beam across the surface of the geometry and capturing speckle images, which can be accomplished within a few seconds. This can be particularly helpful in longitudinal studies of brain tissue hemodynamics. This is because any subsequent imaging session involves only capturing a few speckle images in the desired time intervals and processing such images offline to extract high-resolution blood flow maps.

We previously showed that assuming tissue VFs instead of intact complex structure can lead to large errors in inferring BFI estimates resulting in large resolution and accuracy degradation.^13^ As such, our reconstruction algorithm proposed in this paper uses the most rigorous and extreme case that includes all vascular structures for proof of concept. However, we view this as a necessary first step toward 3D blood flow imaging. Once the method has been validated, we will investigate the accuracy with which the vascular structure needs to be known. Particularly, it has been shown that vascular anatomy in a certain region of the brain is consistent within a given species.^47^^–^^49^ As a first step we will explore replacing the smaller vasculature and capillary networks with representative statistical vascular models. This optimization, if successful, relaxes the requirement for high-resolution imaging of the structure and reduces the computational complexity. Therefore, there are some parallels between our methodology and that of diffuse optical tomography reconstruction methods that require a full anatomical MRI scan.^50^^,^^51^ Acquisition of the MRI is a significant, time-intensive, and costly constraint. However, it may be possible to use a more generalized anatomical model in place of a subject-specific MRI scan. Similarly, it may be possible to use a generalized vascular structure to extract depth-resolved blood flow rather than a subject-specific vascular structure in the future.

Another limitation is that the time required for reconstruction makes dynamic 3D imaging currently impractical. However, most 3D inverse problems are not capable of real-time imaging due to significant time and resource requirements, but they do provide high-resolution results. In our case, the combination of vascular flow reconstruction accuracy, resolution, and FOV is well beyond what has been reported in the literature previously. Furthermore, there are many uses of cerebral blood flow imaging that do not require real-time imaging or measurements over many time points. Chronic studies that analyze cerebral blood flow changes over long periods of time typically only require a single image at each measurement. In such applications, the time required for the reconstruction would be quite reasonable.

Finally, although the simulation times reported here have been optimized for parallel and distributed computing, as discussed earlier in Sec. 4.3, the reconstruction algorithm can be optimized for processing on GPU. Our analysis showed that reconstruction time was ∼3× longer on a single GPU as compared with the 200 cores utilized on a distributed computer system. In subsequent studies, we will explore the effect of vascular structure in conjunction with further optimization for processing on GPU to significantly reduce the reconstruction times.

## 5 Conclusion

We have presented a novel computational method for high-resolution imaging of blood flow maps in complex tissue over a large FOV for a known structural prior. Our simulation results indicate high fidelity reconstruction of blood flow maps down to capillary level beyond 500-μm depth if the 3D geometry is known or assumed. The unique property of our proposed method lies in its object-based reconstruction capability. Hence the resolution is dictated by the spatial resolution of the vascular objects (strands). For the geometries presented, the resolution of the smallest individual objects (capillary strands) is <10  μm.

A combination of PMC-based acceleration methods in conjunction with advanced optimization algorithms implemented for large-scale inverse problems, and efficient parallelization and vectorization, allowed for feasible reconstruction time (on the order of 10s of mins). We note that in its current state, our proposed reconstruction algorithm is only complimentary to high-resolution imaging modalities such as OCT or 2/3PM as it allows for high temporal resolution imaging of hemodynamics over a large FOV. Once a high-resolution structural image is captured, our reconstruction algorithm only requires a few LSCI images for each illumination source, captured through a camera, to reconstruct the blood flow map at a given timestamp. We foresee that our proposed methodology can particularly have a high impact in enabling high-resolution visualization of hemodynamics during neural functional activation studies.

The recent advances in fast, high-resolution PMC-based optical tomography methods enabled through hybrid and mesh-based MC simulation platforms,^28^^,^^52^^,^^53^ can perhaps pave the way for fast and noninvasive extraction of the structural geometry needed in our reconstruction problem only requiring very simple instrumentation.

As a future direction, we will also examine using mesh-based MC models combined with learning algorithms for more efficient selection of detectors and source patterns to speed up our reconstruction times significantly.
