# Supplementary material for: High-resolution three-dimensional blood flow tomography in the subdiffuse regime using laser speckle contrast imaging
Source: J Biomed Opt. 2022 Mar 31;27(8):083011. doi: 10.1117/1.JBO.27.8.083011 (PMC8968074; doi:10.1117/1.JBO.27.8.083011)
Supplement: Supplementary file 1 [file JBO_027_083011_SD001.pdf]

High-resolution three-dimensional blood flow tomography in the sub diffuse regime using laser speckle contrast imaging: supplemental materials

Chakameh Z. Jafari,[a] Samuel A. Mihelic,[b] Shaun Engelmann,[b] and Andrew K. Dunn[b,*]
[a] Department of Electrical and Computer Engineering, The University of Texas at Austin, Austin Texas 78712, USA
[b] Department of Biomedical Engineering, The University of Texas at Austin, Austin Texas 78712, USA
*Andrew K. Dunn, E-mail: adunn@utexas.edu

This document contains supplementary information to "High-resolution three-dimensional blood flow tomography in the sub diffuse regime using laser speckle contrast imaging." We provide discussion on the result of assumed vascular flow direction along vascular centerline, on the simulated speckle contrast images and reconstruction accuracy.

1. Effect of Vascular Flow Direction on the Simulated Speckle Contrast Images

Given that the vectorized vasculature is not a closed network system, our ability to trace the network and derive direction is limited. As such, directions were randomly assigned along the vascular centerlines in the vectorization step. To evaluate the effect of this assumption on our forward model, we generated speckle contrast images by globally randomizing the assumed directions along the centerline of all strand objects in the geometry and compared the results. A different pseudo random sequence $s \in \{-1,1\}^N$, N the size of the total number of strand objects, were generated to be multiplied by $V = [v_1, v_2, v_3, ..., v_N]^T$ before computing the speckle contrast image for each case. Intuitively this results in flipping the direction along the centerline where a -1 is multiplied by the scalar component of the vascular flow v_i .

Figure S1 illustrates the comparison of simulated speckle contrast images for two different randomized directions along the centerline under illumination 1 scheme. As shown, even with global randomization of the flow directions, the difference between the two observed speckle contrast is less than $\pm 5\%$. These results are expected because the amount of decorrelation in the observed speckle contrast is related to the magnitude of flow in each strand object and not the sink or source directionality of the flow.

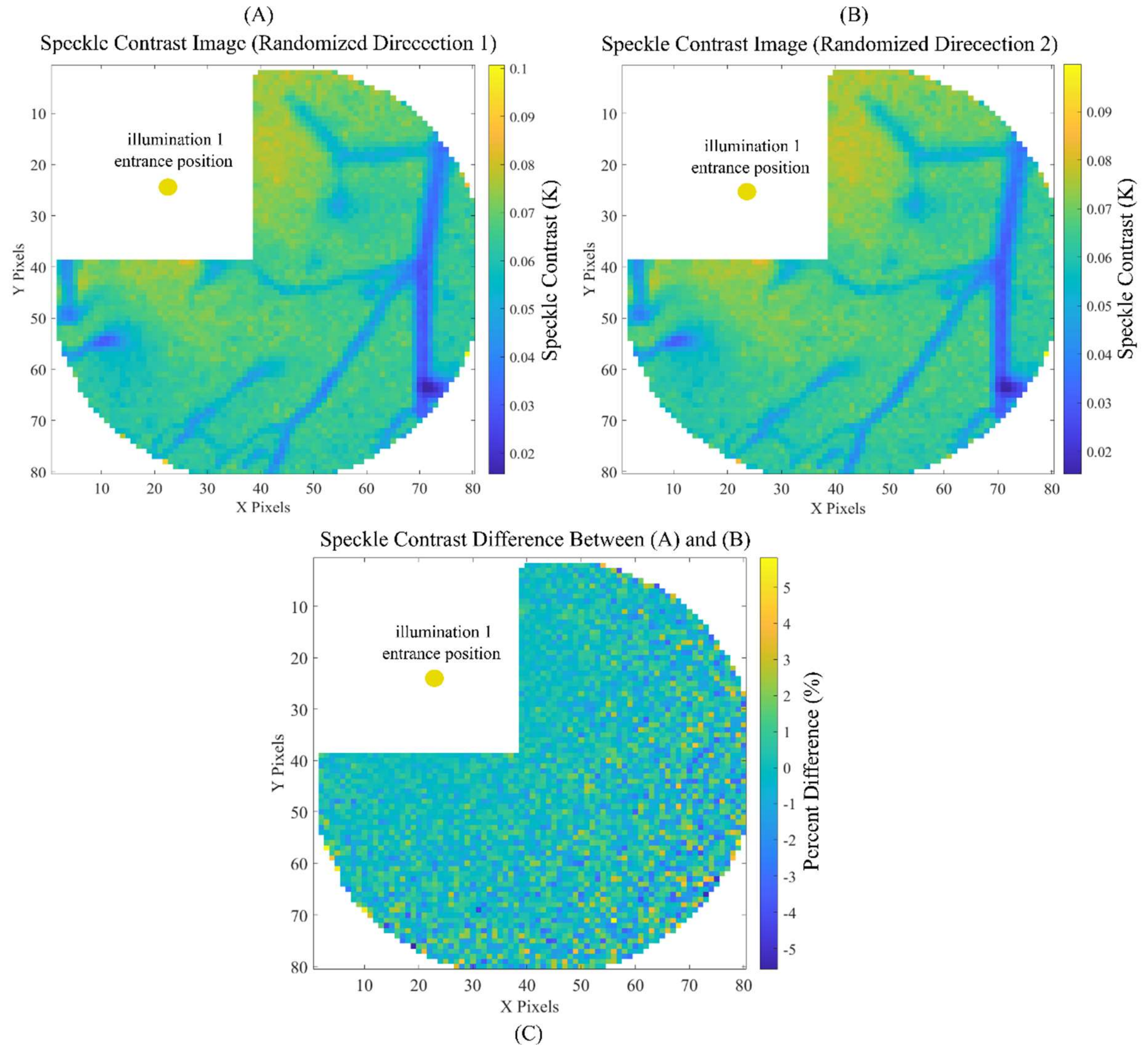

Fig. S1. Comparison of observed speckle contrast images for two globally randomized directions (± 1) along vessel centerlines. (A) Speckle contrast image for randomized direction 1. (B) Speckle contrast image for randomized direction 2. (C) Percent difference between the simulated speckle contrast images (A) and (B). As shown the difference less than $\pm 5\%$.
